# Mercury Accumulation Pathways in a Model Marine Microalgae: Sorption, Uptake, and Partition Kinetics

**DOI:** 10.1021/acsestwater.3c00795

**Published:** 2024-06-10

**Authors:** Isabel Garcia-Arevalo, Jean-Baptiste Bérard, Johannes Bieser, Séverine Le Faucheur, Clarisse Hubert, Thomas Lacour, Bastien Thomas, Daniel Cossa, Joël Knoery

**Affiliations:** † IFREMER, CCEM Contamination Chimique des Écosystèmes Marins, F-44300 Nantes, France; ‡ IFREMER, PHYTOX Physiology and Toxins of Microalgae, F-44300 Nantes, France; § Institute of Coastal Research, Helmholtz-Zentrum Hereon, Max-Planck-Str. 1, 21502 Geesthacht, Germany; ∥ Université de Pau et des Pays de l’Adour, E2S-UPPA, CNRS, IPREM, 64000 Pau, France; ⊥ Université Grenoble Alpes, ISTerre, CS 40700, 38058 Grenoble Cedex 9, France

**Keywords:** mercury, methylmercury, phycosphere, partition coefficient, marine phytoplankton, uptake

## Abstract

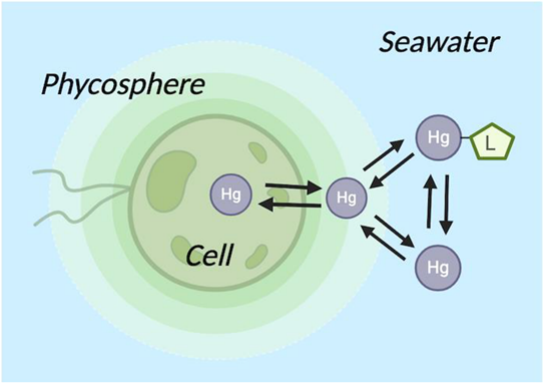

The accumulation of dissolved mercury (Hg) by phytoplankton is the largest concentration step along aquatic food chains. However, the cell uptake mechanisms remain unclear. In this study, the marine haptophyte*Tisochrysis lutea*, a model phytoplankton species, was examined for its interactions with picomolar levels of dissolved inorganic divalent Hg (iHg) and monomethyl Hg (MMHg). For both these Hg species, the study observed their successive sorption and internalization over time, yielding Hg partition coefficients as well as sorption, uptake, and release rates. These results were integrated into a time-dependent, three-compartment model for marine cellular Hg accumulation that included exposure medium, phycosphere, and internalized mercury. Assuming equilibria and pseudo-first-order kinetics between compartments, this study obtained transfer rates of Hg between compartments. The results provide insight into the phycosphere as an intermediate compartment for Hg species accumulation and quantify its role in the internalization of Hg. Ultimately, the new model and its parametrization were successfully applied to literature data showing Hg cellular accumulation in different groups of marine phytoplankton, lending confidence in its robustness and potential contributions to help model the uptake of Hg in the aquatic food web.

## Abstract 2

We quantified experimental parameters governing the observed interactions Hg− phytoplankton and proposed two-pathway Hg cellular accumulation model applicable to different groups of phytoplankton summarizing our understanding of the interactions.

## 1 Introduction

Mercury (Hg) raises a significant global issue due to its highly bioavailable and neurotoxic methylated forms, such as monomethylmercury (MMHg) and dimethylmercury (DMHg).^1,2^ These organic mercury species exist in marine waters and are a product of a delicate balance between their continuous production and degradation, as well as the availability of its inorganic form (iHg).^3−5^ Phytoplanktonic uptake of Hg species plays a pivotal role in marine food webs, as it serves as the primary entry point for MMHg, which biomagnifies up trophic levels and can become of health concern due to its neurotoxicity.^5^

Hg abundance and bioavailability in water are shaped by removal and remobilization processes, leading to Hg redistribution between dissolved and particulate phases.^6^ This “regenerative scavenging” transfers dissolved Hg from surface waters into the particulate phase through biological uptake and releases it as particles degrade.^6−8^ Various factors, including dissolved organic matter (DOM), reduced sulfur compounds, and nutrient flow, govern Hg bioavailability for phytoplankton, itself an important class of marine particles.^9−12^ Consequently, better understanding the distribution of mercury species in the presence of phytoplankton is crucial for improving our understanding of the oceanic Hg distribution.

The empirical partition coefficient (*K*_d_) is extensively used to quantify the scavenging of dissolved elements in natural waters.^13^ However, the magnitude of *K*_d_ for Hg remains uncertain and is a major source of uncertainty in marine Hg cycle models,^14−17^ since interactions between Hg and other solutes, especially DOM, significantly impact *K*_d_.^18^ Notably, *K*_d_ decreases with increasing organic matter.^19^ We hypothesize that phytoplankton, a dominant type of particulate organic matter in the open ocean, plays a central role in determining the oceanic *K*_d_ values.

Phytoplankton’s growth and phenology are key to quantifying the scavenging of Hg species by marine particles.^20^ The intake of metals in phytoplankton cells results from both passive and active uptake mechanisms and is further constrained by bioavailability and abundance of metals.^21−24^ While studies have investigated Hg uptake by freshwater phytoplankton and its trophic transfer in freshwater ecosystems,^11,22,25,26^ research on marine environments has primarily focused on a limited number of species of different phytoplankton groups, showing contrasting results.^10,21,27^ Metal uptake by algal cells generally involves initial surface adsorption and the subsequent transport into intercellular pools.^28^ However, the varying definitions of phytoplanktonic marine particles have led to a wide range of results and made predictions of Hg bioavailability for phytoplankton challenging. Particularly, the operationally defined exterior of the cell for Hg uptake usually differs between the field and laboratory.^29,30^

Understanding the interactions between mercury and phytoplankton is fundamental for modeling the Hg behavior in oceans. To improve these models, comprehensive information on Hg speciation, partitioning, and bioaccumulation dynamics is essential.^14^ However, the mechanisms of algal Hg uptake and the factors regulating this process in aquatic ecosystems are still not well-defined.^16,24,31^ Even though there is a general understanding of phytoplankton Hg uptake, an intercomparable approach to how fast and to what degree this mechanism takes place is still lacking. Indeed, it has been shown that it depends on the physical and biological characteristics of individual phytoplankton species (*S*/*V* ratio, phenology), and on environmental conditions (dissolved organic carbon (DOC), *T*°, Salinity, nutrients).^10,23,32,33^ Furthermore, the phycosphere, the microenvironment surrounding plankton cells^34^ where the effects of algal exudates and its associated microorganisms are significant,^35,36^ has had limited examination in Hg-phytoplankton dynamics.^33^ The consideration of this intermediate reservoir might influence various results of Hg species uptake by marine phytoplankton.^37^

To improve our understanding of how phytoplankton, as marine living particles, influence dissolved marine mercury behavior, this study aimed to quantify, at environmentally relevant concentrations, the interactions between two mercury species, iHg and MMHg, and a model phytoplankton. Particularly, this study is designed to assess the role of the phycosphere as an intermediate compartment, in the uptake of Hg species by marine phytoplankton. Sorption equilibria and uptake rates of both Hg species by *Tisochrysis lutea* were determined, distinguishing between adsorption and internalization of both Hg species. These parameters were then used to create time-dependent scenarios of mercury distribution within marine phytoplankton, highlighting the role of the phycosphere as an intermediate accumulator for Hg species prior to the internalization of Hg.

## 2 Materials and Methods

### 2.1 Plankton Culture Conditions

The algal strain *T. lutea*, previously named *Isochrysis affinis galbana* (Tahiti isolate), was provided by the Culture Collection of Algae and Protozoa (CCAP 927/14). The haptophyte *T. lutea* was chosen because of its ocean-wide distribution, ease of culture and physical features to be used as a reference phytoplankton for the uptake model.^38,39^ This planktonic algae was grown under axenic conditions in acid-cleaned, borosilicate glass bottles containing filtered (0.2 μm), autoclaved natural seawater (35 psu, coast of Saint-Malo, France) amended with the f/2 growth medium.^40^ The cultures were aerated by bubbling 0.22 μm-filtered air. Throughout the experiments, the cultures were held at 22 ± 2 °C and subjected to continuous irradiance levels (300 and 100 μmol m^–2^ s^–1^ measured in the front and at the back of the bioreactor, respectively), continuous axenic conditions were verified by Sybr green staining and fluorescence microscopy.^41^

### 2.2 Mercury Exposure Experiments

#### 2.2.1 Preparation

**Figure 1:**
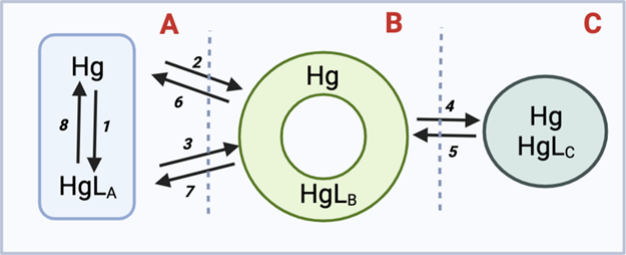
Schematics of Hg species interactions with the model phytoplankton. The three studied compartments are analytically resolved: dissolved culture medium (A), phycosphere (B), and cell interior (C). The arrows are numbered to identify the processes used in eq 2. For each compartment, Hg corresponds to the Hg species as a free ion, and HgL_*x*_ is the ligand-complexed Hg. It should be noted that Hg and HgLb, and that Hg and HgLc are not differentiated analytically.

Exposure experiments were initiated with a monospecific *T. lutea* inoculum (ca. 1 L) dispensed in a culture flask containing ca. 8 L of filtered and autoclaved seawater. The f/2 enrichment complex was not added to the culture flask to minimize interference from its EDTA with plankton-produced ligands during the experiments. Nevertheless, ca. 60 nM EDTA was carried over from the inoculum of the mother culture. Once the cell density reached 10^6^ cells mL^–1^, signaling the near onset of the stationary phase, established from previous *T. lutea* growth curves (Figure 1 SI), a solution containing the studied Hg species was added to meet the target concentration of 5 ng Hg L^–1^ (25 pM), and the culture was shaken until homogeneity. Immediately after the Hg addition, *>* 2 L culture aliquots were distributed into four bioreactors, one for each exposure time step (Control, T2, T8, T24 h). The remainder of the culture was used as the initial time point (T0.5 h) and processed immediately. The two experiments, one for every Hg species, were limited to a 24-h period to minimize changes in the composition of the culture and duplicated to assess overall experimental variability. At each time point, algal cell density and biovolume were measured using a Beckman Coulter Multisizer 3 cell counter (aperture size 100 μm, analytical volume 100 μL, sample dilution 100×) according to the manufacturer’s method specifications.

For each experiment, the bioreactors were connected to a 0.22 μm-filtered air supply with gold traps placed both at the inlet and outlet. The stream of air ventilated only the headspace above the algae exposure medium to collect dissolved gaseous mercury (DGM; gaseous elemental and dimethyl mercury) that could have degassed from the culture.

#### 2.2.2 Culture Sample Processing

At each exposure time, the phytoplankton cells exposed to Hg were concentrated into a pellet by mild centrifugation (2500*g*, 15 min, 4 °C). Given that the centrifugation conditions were kept mild to prevent cell rupture, it allowed some (<1%) of the *T. lutea* to leave the pellet and return to the supernatant. For an accurate inventory of Hg in the dissolved and particulate phases, an aliquot of the supernatant was filtered through previously combusted GF/F (0.7 μm) to remove particulate Hg from cells that had strayed from the pellet from the dissolved compartment. Filtered and unfiltered centrifuged supernatants were acidified to 0.5% v/v with 11 N HCl (trace metal grade), and refrigerated until dissolved Hg species analyses (<1–2 days). Reported dissolved mercury levels were those of the filtered supernatant.

Particulate adsorbed Hg was quantified by resuspending a known portion of the centrifugation pellet for 5 min in 100 mL of an 8 mM cysteine solution,^30,33^ shaking and then centrifuging it again at 2500*g* for 15 min. This scheme was previously used by Cossa (1976) for cadmium to remove any Hg adsorbed to the outer cell membrane.^42^ The cysteine supernatant accounted for particulate adsorbed Hg, and particulate absorbed Hg, hereafter internalized Hg, is the Hg fraction remaining in the cysteine-rinsed pellet.

Each of the three main compartments (dissolved phase, particulate adsorbed, and particulate internalized phase) was analyzed for Hg species, as well as any Hg retained on bioreactor walls by acid rinsing the bioreactor walls (4 M HCl), and the gaseous efflux measured from the outlet gold traps.

### 2.3 Mercury Analyses of the Different Compartments

These experiments used monospecific mercury solutions of inorganic mercury (iHg) and MMHg was used. Hereafter, iHg refers to all forms of inorganic Hg, (i.e., Hg^2+^ in its ionic form, hydroxyl-, chloro-, hydrochloro-Hg, and Hg^2+^ bound to DOM), while MeHg to the sum of the methylated forms of Hg, (i.e., ionic MMHg and gaseous DMHg, as well as MMHg complexed with available ligands), since our analytical scheme is unable to discriminate them. The obtained mass balances suggest that the latter species was not significant.

#### 2.3.1 Dissolved iHg

iHg was quantified using a technique based on the US-EPA 1631 method implemented on a lab-built apparatus.^43,44^ Briefly, samples were treated using 100 μL of a bromine monochloride (0.2M) solution in 9 N HCl to oxidize all of the Hg species to Hg(II) in a 60 mL PFA bottle. Then, an aliquot of the mineralized sample was placed in a PFA sparging vessel with an excess of SnCl2 solution in order to reduce quantitatively Hg(II) to gaseous Hg(0), which is then stripped from the solution using an argon stream. The evolved gases pass through a gold-coated sand trap which amalgamates and retains Hg(0). After Hg(0) is quantitatively extracted from the sample, the Hg retained on the trap is released by thermal desorption (600 °C), and then quantified by cold-vapor atomic fluorescence spectrometry (CVAFS) using a Tekran 2500 detector. Standard solutions of Hg(II) at 999 μg mL^–1^ (SPC Science) were used for external calibration. Accuracy and response stability over time was verified every fifth run using an aliquot of ORMS-5 certified reference material (CRM) produced by the National Research Council of Canada with recoveries of 100.2 ± 3.1% (1 SD, *n* = 8). For analyses of a 40 mL sample, the limits of detection (LOD) and quantification (LOQ) are 0.3 and 6 pg L^–1^ based on a 15 pg L^–1^ solution and a blank sample, respectively.

#### 2.3.2 Dissolved MeHg

Monomethyl mercury was analyzed as MeHg using an analytical technique based on hydride generation which was adapted from Cossa et al. (2009).^45^ The concentration was determined by using a lab-built instrument and coupled to a CVAFS instrument (Tekran 2500). In brief, an acidified, 40 mL sample is added with a sodium borohydride solution (NaBH_4_ dissolved in Milli-Q water at 1% w/v at 250 μL min^–1^) and sparged with helium gas (140 mL min^–1^). This treatment converts Hg species into their volatile hydrides, which are then swept into a cryogenic/gas chromatographic trap. The trap (OV3–15%, silicone-coated Chromosorb) quantitatively captures the exsolved Hg species, until they are sequentially released as temperature progressively increases (60 °C, He at 20 mL min^–1^), converted into Hg(0) (quartz tube at 800 °C), and detected via CVAFS. Calibration used MMHg chloride (99% purity, Strem Chemicals) standard solutions. Due to the lack of a CRM for dissolved MMHg, and instrument sensitivity drift, sample analyses are bracketed by analyses of a standard every third run. Based on the standard deviation of repeated analyses of a 4 pg L^–1^ solution, the LOD is 25 pg L^–1^, and the LOQ is 75 pg L^–1^ for a 40 mL sample size. This analytical scheme determines MMHg and any gaseous DMHg that may have been present in the sample before acidification, and thus analytical results are referred to as methylated mercury, MeHg. Intercomparison of this method with isotope dilution inductively coupled plasma sector field mass spectrometry (ID-ICP-SFMS) conducted on fM levels of MeHg in seawater yielded a coefficient of variation values better than 10%.^46^

#### 2.3.3 Particulate iHg

iHg in freeze-dried centrifugation pellets or in tared, oven-dried GF/F filters was determined as total Hg using an automatic atomic absorption spectrophotometer AAS (model AMA-254, Altec), according to the protocol described by US EPA (2007).^47^ The instrument was externally calibrated with Hg(II) standard solutions (999 μg mL^–1^, SPC Science). The LOD was 0.5 ng, corresponding to 0.005 μg g^–1^ for a 100 mg sample. LOQ was 1.5 ng and 0.015 μg g^–1^. Daily QA/QC was performed with triplicate analyses of sediment CRM (MESS-4) with recoveries of 92 ± 7%, (1 SD, *n* = 20), while sensitivity was verified with freeze-dried plankton CRM (BCR 414) with recoveries fluctuating 81.4 ± 2.2% (1 SD, *n* = 20).

#### 2.3.4 Particulate MeHg

For the determination of MeHg in freeze-dried pellets, a liquid–liquid extraction of organic Hg was performed before AAS analyses, following a method modified from Azemard and Vassileva (2015).^48^ First, the sample was placed in a 60 mL, Oak Ridge-type, PFA centrifuge tube and digested with HCl (25% v/v) for 60 s at ambient temperature. Then, MeHg was quantitatively extracted into an organic phase (toluene) for 1.5 min. Finally, organic Hg was back extracted into an aqueous phase using a 2 mM thiosulfate aqueous solution. An aliquot of the latter was analyzed as total Hg using AAS. Each batch of 14 samples was processed with 1 blank and 3 CRMs to obtain robust procedural blank and recovery data. Bracketing during AAS runs was used to monitor the instrument sensitivity drift and to calculate the final concentrations in the samples.

For these analyses, the LOD was 0.1 ng and the LOQ was 0.5 ng Hg. For our sample size, this corresponds to 0.005 and 0.025 μg g^–1^. The accuracy of the procedure was monitored using duplicate analyses of CRM NIST 1566b with each batch of samples. The liquid/liquid extraction efficiencies averaged 86.2 ± 2.9% (1 SD, 10 batches). At the analytical conditions summarized above, and following GUM/Eurachem guidelines, the relative standard uncertainty (*k* = 2) of our analytical results of freeze-dried plankton was 9.6% at the level of 0.13 μg g^–1^ of MeHg.

### 2.4 Ancillary Parameters

Particulate carbon and nitrogen concentrations were measured by using an Organic Elemental Analyzer (Thermo Scientific). A TOC meter (VSH model, Shimadzu) was used to measure the concentrations of total, organic, and inorganic carbon and nitrogen (TOC, TIC, TON) in both the culture medium, the unfiltered and filtered supernatants, to account algae produced DOM (cellular exudates) in the form of dissolved organic carbon (DOC) concentrations. All measurements were conducted according to the EPA protocol 415.3 method specifications.

### 2.5 Data Assimilation into a Three-Compartment Model

In order to represent the pathways of Hg species transfer between three physical compartments (the culture medium, phycosphere, and the interior of the cell), which have been operationally defined using the sample processing and analytical schemes described before, we use the conceptual model that comprises three compartments and four-site interactions presented in Figure 1. The proposed model is adapted from cellular metal uptake models and accounts for the presence of complexed Hg in the dissolved and adsorbed phases.^49^ As our experimental data did not differentiate between complexed and free Hg forms, HgL_*x*_ represents Hg bound to ligands from each compartment, including hydroxyl-, chloro-, hydrochloro-Hg, and organic phytoplankton-produced ligands. All complexes are lumped into a single ligand L, even though the form and proportions might differ in each compartment.

The model uses reversible equilibria between the sites. Within this representation, the conservation of mass of added spiked Hg at any time *t* in the different compartments of the culture is represented by
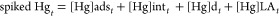
1where [Hg]_*t*_ is expressed as ng of Hg in 1 L of bulk culture medium for each compartment, whereas [Hg]ads is the concentration of Hg adsorbed to the phycosphere of *T. lutea*, [Hg]int is internalized Hg, [Hg]d is dissolved Hg in its free ion form, and [Hg]LA is Hg associated with any ligand within the dissolved phase (compartment A).

The following equations set describe the changes in Hg concentration in the different studied sites:
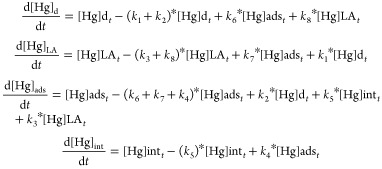
2where *k*_*n*_ represents the different rate of transfer of Hg between the compartments and each number *n* is identified in Figure 1. Transfer of Hg in each compartment over time was described in the system of first-order differential eqs (eq 2). Model coefficients were obtained from our available measurements of initial and equilibrium concentrations and from iteratively optimizing the missing rates of transfer (*k*_*n*_) within predefined ranges. Assuming that these transfers are pseudo-first-order (PFO) processes, the concentrations can be described by the coupled equations, such as
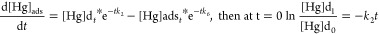
3where the forward reaction rate *k*_2_ refers to adsorption to cell walls, and its back-reaction rate *k*_6_ refers to desorption from the cell walls. These equations apply for iHg and MMHg since we did not observe interconversion of Hg species or loss to walls or volatilization was minimum (see below). Assuming that the partition between the particulate and dissolved phases was at equilibrium, we obtained the back-reaction rate constants. Indeed, at T24 when the change over time of the amount of Hg in the particulate phase was negligible, we obtain
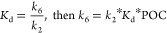
4where POC refers to particulate organic carbon concentrations in kg L^–1^, and *K*_d_ refers to the partition coefficient in L kg^–1^. Hg partition at equilibrium (*K*_d_) between the medium and the cell phycosphere, the medium and the cell internalization, and the cell phycosphere and cell internalization were determined using the following equation:
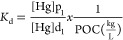
5Once the corresponding rates constant and partition were obtained as summarized above, the set of equations describing the change in Hg availability (eq 2) was numerically integrated using Scientific Python Development Environment Spyder 4.5.2 (Python 3.9.14) and a predefined range of rates to optimize the best fit to the data based on the lowest mean square error (MSE). Significant decreases in MSE between successive iterated solutions and experimental data were determined through paired *t-*test, with a significance threshold of *p* = 0.05. The deriving assumptions, full equations, and fitting of error-minimizing procedures of the computations are described in detail in SI (Appendix A).

Finally, to facilitate the intercomparison between our experimental data and other data reported in the literature, we also computed our obtained mercury species distributions and rates with respect to the concentrations of total suspended matter (SPM kg L^–1^), particulate organic carbon (POC, kg C L^–1^), cell abundance (cell L^–1^), cell surface (μm^2^ L^–1^), cell volume (μm^3^ L^–1^), and dissolved Hg (ng L^–1^) in the culture.

## 3 Results and Discussion

### 3.1 Distribution of iHg and MMHg over Time

**Figure 2:**
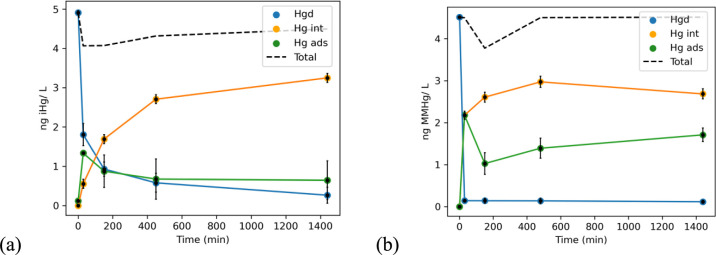
Temporal distributions of the recovered iHg (a) and MeHg (b) in each of the three compartments, reported in ng of Hg per 1 L of culture volume. Error bars represent one SD for the experimental duplicates. The dotted line is the numerical sum of the Hg species recovered at each time point.

To enable meaningful comparisons despite variations in culture volumes and cell densities (6–9%), we normalized and reported here the Hg inventories in each compartment to the quantities expected for 1 L of bulk culture. Recoveries averaged 93 ± 4.1% and 96 ± 7.2% for iHg and MMHg experiment, respectively. The distributions of Hg in the three compartments are shown in Figure 2, exhibiting their time-dependent dynamics, and the overall coherence of the experimental results.

For iHg, its progressive association with the particulate fraction (i.e., adsorption and internalization into plankton cells) was accompanied by corresponding decreases of its dissolved concentrations in the medium, ending with 94% of the recovered iHg being associated with plankton after a 24-h period. Importantly, the data show a temporary maximum of iHg in the B compartment, the phycosphere. This maximum is consistent with a faster but temporary adsorption of iHg, compared to its internalization within the cells. After 24-h exposure, the recovered iHg spike distributes as 6 ± 1.1% dissolved, 18 ± 0.3% adsorbed, and 76 ± 2.6% internalized Hg. The amounts of recovered DGM and glassware mercury are negligible at <1%. Indeed, recovered DGM increased slowly over time but never exceeded 0.4% of the initial iHg added, and the glassware-adsorbed iHg was <0.2% at all time points. This results in a maximum reduction rate of 1.7 × 10^–4^ h^–1^ for iHg.

For MMHg exposure, the particulate fraction of MMHg increased rapidly and reached 90% within the first 30 min. Similarly to iHg, a distinct temporary maximum of adsorbed MeHg was observed. At the end of the experiments, the distribution of the spiked MMHg was 2.5 ± 0.8% dissolved, 37 ± 3.6% for adsorbed, and 59 ± 2.6% for internalized MMHg. Like with iHg, DGM concentration increased throughout the 24 h but never exceeded 0.7% of the total MMHg added (maximum reduction rate of 2.9 × 10^–4^ h^–1^ for MeHg), while the glassware-adsorbed MeHg was <0.16% at all time points. This validates the assumption that the spiked MMHg and the analyzed methylated species (=MMHg + DMHg) are equivalent.

Our experimental results indicate that after a few hours of exposure, most iHg has been internalized by *T. lutea*, in contrast to adsorbed and internalized pools of MMHg that are evenly distributed. This finding contrasts with previous studies, where MMHg was found predominantly internalized and bound to cytoplasmic proteins (∼60%), and where inorganic mercury remained outside the cells (∼90%).^21,33,50^ These differences may be due to experimental conditions that make more detailed comparisons difficult. Indeed, the previous experiments were performed at Hg species concentrations that were many orders of magnitude higher (i.e., nM to μM levels) and at different cell densities, DOC conditions, and EDTA levels. We can hypothesize that at elevated levels of Hg, adsorption sites of the phycosphere and membrane surface could be saturated by the added Hg, changing the adsorption equilibria and transfer rates. However, in the present study, the dynamics of the adsorbed Hg pool suggest that the phycosphere was not overwhelmed by the added Hg, even though the Hg supply to its surface was not diffusion-limited (calculation in Appendix B SI).

### 3.2 Kinetics of iHg and MMHg Cellular Accumulation

To quantify the rates of concentration change involved in biotic interactions and transformation of Hg, we used both the initial and final concentrations of the three compartments and the numerical integration scheme described above. Thus, the following are the results of the kinetic parameters that were obtained, based on our experimental measurements and the cellular accumulation model results.

#### 3.2.1 Partition Coefficients

The equilibrium partition coefficients *K*_d_ were calculated using dissolved Hg concentrations with either suspended particulate matter (SPM) or particulate organic carbon (POC) concentrations and are reported in Table 1. The concentrations are those observed at “equilibrium” i.e., after 8 h of exposure, even if they vary slightly in each of the three compartments. Table 1 shows the resulting *K*_d_ representing the partition between dissolved Hg and the three analyzed forms of particulate Hg, i.e., total particulate, adsorbed, and internalized Hg. SPM was computed by multiplying cell density and average single cell dry weight (34 pg cell^–1^) taken from Cañavate et al., (2020).^51^ After 24 h of exposure, we observed that iHg is less particle-reactive than MeHg by nearly a factor of 10. Also, more Hg is an internalized Hg species than an adsorbed Hg species, even more so for iHg than for MMHg.

**Table 1:**
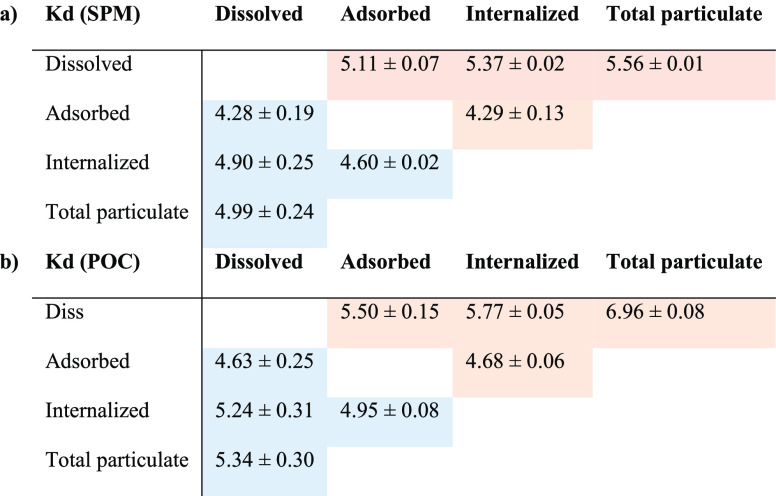
iHg (Blue) and MeHg (Red) Partition Coefficients (log L kg^–1^) for Adsorbed, Internalized and Total Particulate vs. Dissolved Hg, and Internalized vs. Absorbed Hg, Calculated Relative to (a) SPM (kg L^–1^)^a^, and (b) to POC (kg C L^–1^)^a^

a Uncertainties are computed from 1SD between data collected at times T8 to T24 h.

The study provides an internally consistent set of partition coefficients for the distribution of iHg and MMHg between compartments of a phytoplankton culture. Their field-derived equivalent by Cui et al. (2021) showed that particulate organic matter (POM) is an important contributor to overall *K*_d_ values.^6^ The *K*_d_ values from the present work (Table 1) provide an endmember value of *K*_d_’s for particulate organic carbon. We observed that MMHg *K*_d_ (5.6 log L kg^–1^) is similar, yet higher than iHg *K*_d_ (5.0 log L kg^–1^). Nevertheless, the values of *K*_d_ for Hg from three ocean basins, as seen in Figure 2 SI, are higher yet consistent than those found in the phases represented in this study.^6^ Indeed, when taking into account the decrease in *K*_d_ values as SPM increases,^6,8,52^ the *K*_d_’s from this study can be recalculated using midocean SPM, mercury concentrations, and the ratio of cell density^53,54^ to open ocean dissolved Hg. Using cell densities appropriate for the open ocean, we then obtain values of 6.59 and 6.94 log L kg^–1^ for iHg and MMHg partitions, respectively. The latter values are well within the range of open ocean field studies.^6,8,55^ It should be kept in mind that in contrast to the organic particles that were experimented with in this study, open ocean-collected plankton is enriched in other particles, often mineral-rich clays, which may have distinct mercury adsorption characteristics. Likewise, even though there are limited studies focusing on the partition distribution of MMHg in marine environments, we compared our results with laboratory and field results. We found that previously recalculated *K*_d_ values for MMHg (6.94 log L kg^–1^) are higher than the average values (5.9 log L kg^–1^) reported by Allison and Allison (2005) for SPM and surface water, and (5 log L kg^–1^) Tesan-Orubia et al. (2023) for fraction filtered SPM (2.7–20 μm), but very similar with *K*_d_ from the present study conditions (5.6 log L kg^–1^).^6,44,56^

#### 3.2.2 Dynamics of iHg and MMHg Sorption by *T. lutea*

The experimental parameters summarizing PFO rates of Hg interactions between the medium, phycosphere, and cell interior are given in Table 2. For the transfers where measurements are not available, rates were obtained through the minimization of MSE between collected data and results of computations of the three-compartment and four-site transfer model (Figure 1). Complete details on the definition of the transfer rate constants for each Hg species can be found in Appendix A SI.

**Table 2:** 4-Site Model Transfer Rate Constants of Hg Species Accumulation by *T. lutea* by the Proportion of Hg Transfer Computed on the Bases either of Volume of Culture, of Cell Density, or of Cell Biomass^a^

				per volume (h^–1^)		per cell (cell^–1^ h^–1^)		per biomass (mgC^–1^ cell ^–1^ h^–1^)	
# *k*	direction			MeHg	iHg	MeHg	iHg	MeHg	iHg
1	diss	→	L	7.80	3.00	2.73e-09	8.24e-10	1.88e-05	5.68e-02
2	diss	→	ads	1.85	0.15	6.48e-10	4.22e-11	4.47e-02	2.91e-03
3	L	→	ads	7.79	5.36	2.72e-09	1.47e-09	1.88e-01	1.02e-01
4	ads	→	int	3.27	0.71	1.14e-09	1.95e-10	7.87e-02	1.34e-02
5	int	→	ads	2.08	0.15	7.28e-10	4.20e-11	5.02e-02	2.90e-03
6	ads	→	diss	4.80e-02	4.72e-02	1.68e-11	1.30e-11	1.16e-03	8.94e-04
7	ads	→	L	0.54	1.86	1.87e-10	5.11e-10	1.29e-02	3.52e-02
8	L	→	diss	0.55	1.68	1.92e-10	4.61e-10	1.32e-02	3.18e-02
total	diss Hg	→	cell	9.65	5.52	3.37e-09	1.51e-09	0.23	0.10
	cell	→	diss Hg	0.58	1.91	2.04e-10	5.24e-10	1.41e-02	3.61e-02

a This table reports on the forward and back transfer rate constants obtained from the solutions to empirical pseudo-first-order equations describing the dissolved, internalized, adsorbed, and total particulate Hg species; the indices of *k*'s identify the process shown in Figure 1.

In order to properly compare the magnitude of sorption of different Hg species by *T. lutea*, we distinguished cellular inventories of iHg and MMHg between their adsorbed and internalized compartments. Here, we observed that MMHg uptake (7.87e-02 mgC^–1^ cell^–1^ h^–1^) was 6 times faster than iHg uptake (1.34e-02 mgC^–1^ cell^–1^ h^–1^), consistent with previous studies.^57,58^ For iHg uptake by phytoplankton species, the uptake rates based on cell density reported in nutrient-deficient, modified high salt medium by *Chlamydomonas reinhardtii* (3.01e-8 ^1^cell^–1^ h^–1^ (pH 7, Hg(OH)_2_)) are lower in this study (1.95e-10 ^1^cell^–1^ h^–1^) and for other freshwater phytoplankton.^22,59^ Likewise, the MMHg uptake rate constants were found to be three and seven times higher than previous studies using *T. lutea* (0.16 μg g^–1^ h^–1^ vs 0.5 and 1.2 μg g^–1^ h^–1^) but much lower than those found for a diatom species with a similar aspect ratio (2.61e-8 ^1^cell^–1^ h^–1^ vs 3.01e-6 ^1^cell^–1^ h^–1^).^10^ The same was observed for 5 other groups of species of phytoplankton reported when exposed to MM^203^Hg radiotracer.^10^ It is important to note that the comparison is made with an uptake rate computed from the latter study (uptake during the first 4 h of nM levels of exposure). It therefore underestimates the uptake values obtained by using our model (1.49e-7 ^1^cell^–1^ h^–1^) closer to the uptake by another species of coccolithophore.^10^ Analogous results are obtained when computing with respect to cell density, cell surface, or cell volume (Table 3 SI).

#### 3.2.3 iHg and MMHg Cellular Accumulation Model

Once the kinetic parameters were obtained based on our experimental measurements and the best-fit rates (see Appendix A SI for details), the next step was to demonstrate that the rate of change of Hg concentrations in each compartment can be used to accurately represent the global interactions of Hg species with *T. lutea*.

**Figure 3:**
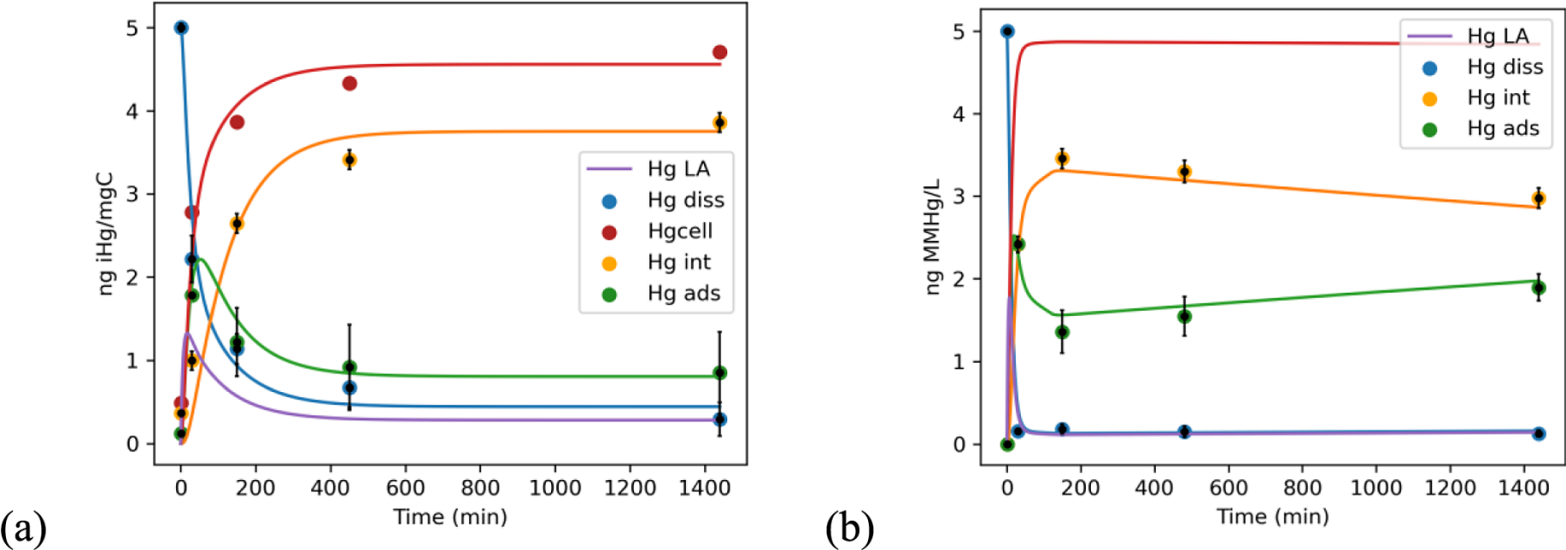
Proportion of Hg recovered per mgC of *T. lutea* in 1 L (circles) and modeled concentrations (lines) of Hg in the dissolved, particulate adsorbed, and particulate internalized phases using the four-site model, for iHg (a) and MMHg (b). The integration parameters are given in Table 2. Modeled dissolved Hg corresponds to Hg from dissolved Hg-LA and Hg-d sites, as Hg complexes were analyzed together with Hg in its free ionic form.

The three-compartment and four-site conceptual model is given in Figure 1 and the best-fitting parameters are shown in Table 2 SI. The resulting numerical integration and rates obtained for each Hg species, expressed on the basis of nanograms of Hg per milligram of C of the cell, are shown in Figure 3 and Table 2. The forward transfer rates of Hg uptake by *T. lutea* followed the predominant transfers of Hg complexes from the medium to the phycosphere and into the cell, while backward transfer into the medium followed the efflux and desorption of Hg complexes to the medium. As expected, the cellular internalization rate of Hg is higher for MMHg than for iHg. Likewise, there is a higher Hg efflux rate and HgLA adsorption rate for MMHg in comparison to iHg, both resulting in faster uptake of MMHg and a balanced distribution of MMHg between the cell and the phycosphere.

In contrast with iHg, the pool of adsorbed MMHg grew with the increase in algal biomass throughout the experiment (Figure 1 SI), and when >70% of the MeHg was associated with cells, the pool of MMHg grew slower than cell density indicating probable biodilution. Consequently, for MMHg, the best fit of modeled vs observed Hg distribution was attained when accounting for either cell growth or biovolume as a physical change of POC in the numerical integration. In turn, the adsorption was decreased and the internalization rate of MMHg increased on a mg per cell basis, and at the same time showed its impact on the uptake inventories of the studied compartments.

It is important to note that our distinction between adsorbed and internalized amounts of Hg is experimentally defined (cysteine-rinse) and hence subject to methodological uncertainty. Nevertheless, the results show the physicosphere as a temporary, reversible compartment for Hg species accumulation. They also show that the phycosphere has an effect on the difference in Hg species internalization.

#### 3.2.4 Hg Internalization

Since there are limited studies considering phycosphere adsorption in Hg uptake dynamics, we can compare results with previous studies using the volume concentration factor (VCF). The VCFs range is higher for MMHg than iHg (*p* < 0.05), highlighting the magnitude of internalization for MMHg. The VCFs from our experiments with iHg and MMHg (4.2 and 4.6), were found to be well within the range of other laboratory studies with freshwater algae (4.2–6.9), but on the lower end of those observed for marine algae (4.3–6.8) obtained at markedly higher Hg concentrations.^10,11,22^ Overall, the present findings at environmentally pertinent conditions are consistent with the mentioned previous field studies, in spite of the operational differences of adsorbed Hg for in situ measurements.^29,60−63^

Because marine phytoplankton is thought to accumulate MMHg mainly by passive uptake from seawater (diffusion) across the cell membrane,^23^ and considering the wide range of sizes and geometry of phytoplankton,^16^ its surface-to-volume ratio (*S*/*V*) is critical for predicting internalization of Hg. Smaller phytoplankton, hence with greater *S*/*V*, are known to maximize nutrient uptake, which should also increase Hg uptake. In this study, the *S*/*V* of *T. lutea* was 1.4 μm^–1^. However, we observed MMHg VFC is various orders of magnitude lower than diatom and coccolithophore species of similar *S*/*V* ratio (1.2–1.4).^10^ This is perhaps due to experimental setup conditions (MMHg exposure, POC, DOC), as well as, the accuracy of computed surfaces and volumes. Nevertheless, when using our model for cellular accumulation and its parameters adapted to those phytoplankton species’ biomass and Hg exposure from Lee and Fisher (2016), we are able to represent the proportion of MMHg taken up by the cells of each phytoplankton group (Table 4 SI and Figure 3 SI). Given that our complexed Hg desorption depends on the POC concentration, the best fit for total cell uptake was obtained when increasing the desorption rate 11.6, 2.6, and 11.9 times for the diatom, dinoflagellate, and cyanobacterium, respectively. This increase factor results from lower POC concentrations in each separate experiment, resulting in different internalizations of Hg. Even though we cannot corroborate differences in internalization, the total cell uptake from these representative marine phytoplankton species was indeed successfully represented with our kinetic model.

Differences between the same study emphasize the importance of taking into account the phycosphere when studying internalization dynamics.^10^ Comparably, differences between the diffusional fluxes of both iHg and MMHg through the phycosphere and Hg internalization by our model microalgae are not proportional to those of other species of phytoplankton.^23^ Thus, the Hg uptake dynamics more likely depend on the phenology of algae, rather than Hg supply to its cellular environment.^20^ Moreover, differences in cell wall thickness and the presence of ultrastructures like scales, frustules, coccoliths, and plates influence the permeability of the cell.^64^ This highlights that more knowledge on Hg uptake pathways and kinetics for phytoplankton species is still needed,^65^ and that it might be linked to species-specific processes. We can speculate that part of the variability could be linked to each species’ phycosphere thickness and its production (e.g., exopolysaccharides that is dependent on cell age, physiological state, or stress) since a thicker phycosphere decreases Hg internalization.^66^

#### 3.2.5 Applicability of the Model

The formation of transient complexes with cell surface ligands, before Hg internalization has been proposed in previous studies.^26,33^ In this study, we include all complexes within the phycosphere, such as DOM. Our findings suggest that they are also key to understanding adsorption to cell surface ligands prior to internalization, as DOC and computed *K*_d__DOC_ contribute to a proper representation of Hg-complexed adsorption rates (Appendix A SI). Comparably, experiments have shown that the differences in the uptake rates are not limited by ligand exchange reactions with MMHg in the water column, but rather the differences in their rate of transfer across the cell wall.^67^ This highlights the importance of considering both dissolved and complexed Hg in the dissolved phase to obtain sorption rates onto the cell’s phycosphere.

Even though in our study we include Hg complexes, we do not differentiate between the pool of ligands (Figure 4 SI). The composition of the dissolved organic complexes is important to define this exchange rate. We can speculate that the algal-derived DOM present in our system may include reduced sulfur and thiols produced by algae, which are likely to be strong ligands for MMHg and iHg,^68−70^ and hence change their bioavailability.^25^ Conversely, for freshwater settings, our internalization rate constant was found to be similar to that of MMHg previously equilibrated with cysteine. Hence, the observed DOC-related complexation of MMHg could be explained by cysteine-like thiols present in the cell exudates.^71^ Nevertheless, even though our model parameters tightly fit with our measurements, more in-depth studies to tackle the DOM dependency within the same setup are still needed.

The rates and partition coefficients that we determined were useful to model the uptake of Hg from sources with mercury levels that are highly variable across short time-scales (e.g., rain events, estuarine mixing, etc.). Nevertheless, iHg and MeHg behave differently. Indeed, iHg adsorption and internalization rates appear constant when normalized to cell density, independent of cell-dependent factors. This would imply that the transfer rates of iHg from transient sources (e.g., rainfall) into cells are dictated by the multiplication of individual cells. In contrast to that of iHg, MMHg dynamics were affected by changes in cell density and biomass. This suggests that MMHg internalization was limited by cell-dependent factors, like changes in the phycosphere thickness or the availability of transmembrane channels to MMHg.^23^

## 4 Conclusions

In general, understanding the degree and speed of absorption and internalization of dissolved mercury species by phytoplankton is crucial for grasping the process by which Hg accumulates within primary producers. The results show how the phycosphere acts as an intermediate compartment for Hg species accumulation, and the effects on Hg internalization are particular to the chemical species considered. We were able to express rates as a function of the physical parameters of the culture, parameters that were measured and controlled (cell density, *S*/*V*, Hg levels). These results, performed at lower Hg exposure than in previous laboratory studies, show that the observed dynamics of the adsorbed Hg species suggest that the phycosphere was not overwhelmed by the amount of added Hg, lending confidence in the field applicability of the study. Furthermore, in contrast to MMHg, iHg adsorption and internalization kinetics appeared to be not limited by cell-dependent factors. These cell-dependent factors might include cell permeability to Hg induced by changes in the phycosphere, such as an increase in thickness or the availability of transmembrane channels to MMHg. Our findings summarized in Figure 1 and Table 2 can be generalized to other phytoplankton species, suggesting that the assumptions behind the model (reversible equilibria between the three compartments, pseudo-first order processes) are valid and can be used for open ocean mercury modeling.

## Supporting Information Available

Additional information on the computation methods for the kinetic modeling (Appendix A), diffusion flux (Appendix B), and further experimental results mentioned in the text (PDF)
