# Supplementary material for: Mercury Accumulation Pathways in a Model Marine Microalgae: Sorption, Uptake, and Partition Kinetics
Source: ACS ES T Water. 2024 Jun 10;4(7):2826–35. doi: 10.1021/acsestwater.3c00795 (PMC11251459; doi:10.1021/acsestwater.3c00795)
Supplement: Supplementary file 1 — ew3c00795_si_001.pdf [file ew3c00795_si_001.pdf]

Supplementary Information

Mercury accumulation pathways in a model marine microalgae: sorption, uptake and partition kinetics

Isabel Garcia-Arevalo[1]. Jean-Baptiste Bérard[2]. Johannes Bieser[3]. Séverine Le Faucheur[4]. Clarisse Hubert[2]. Thomas Lacour[2]. Bastien Thomas[1]. Daniel Cossa[5]. Joël Knoery[1*]*

1 IFREMER. CCEM Contamination Chimique des Écosystèmes Marins. F-44000 Nantes. France

2 IFREMER. PHYTOX Physiology and Toxins of microalgae. F-44300 Nantes. France

3 Helmholtz-Zentrum Hereon. Institute of Coastal Research. Max-Planck-Str. 1. 21502 Geesthacht. Germany

4 Université de Pau et des Pays de l'Adour. E2S-UPPA. CNRS. IPREM. Pau. France

5 Université Grenoble Alpes. ISTerre. CS 40700. 38058 Grenoble Cedex 9. France

Contents **Page**

Appendix A: Kinetic Modelling 2
Appendix B: Mercury diffusion 7
List of Figures 9
List of Tables 13

Appendix A: Kinetic Modelling

The deriving assumptions, full equations and fitting or error-minimizing procedures of the short version of the methods section for *Data assimilation into a 4 sorption-site model* from are described in detail below.

Transfer of Hg in each compartment over time was described in the system of first-order differential equations (Eq. 2). which were integrated using Scientific Python Development Environment Spyder 4.5.2 (Python 3.9.14). Model coefficients were taken from our measurements of initial and equilibrium concentrations. and from iterative optimization of predefined ranges of rates for the conceptual missing reactions.

Considering our operational definition of compartments where Hg-L is not separated. some assumptions had to be taken for the reversible complexation of our spiked Hg to available DOM reaction. The first assumption was to consider DOC as the main ligand present in our bulk medium. following DOM > EDTA > Cl^- concentrations and ligand competition previously studied by Benoit et al. (2001).[1] Nevertheless, for each Hg species a different approach was considered. For iHg, we determined Hg complexed to DOC to use it as a reference for HgL in the dissolved phase; to further compute the Kd_{DOC} at T24 within our experiment results. We took the following equation from Bieser et al. (2023) to obtain Hg_{DOC}[2]:

$$[Hg]DOC_1 = \frac{[Hg]POC_1}{Kd_{POC}*POC_1} - \frac{[Hg]_1}{1+Kd_{DOC}+Kd_{POC}} \quad (1)$$

where Kd_{DOC} is 6.6 (log L kg-1) as depicted in Tesan et al. (2020)[3], and the rest of the parameters in the equation are taken from T24 of our experimental results. Once we obtain [Hg]DOC as a proxy for HgLA, we calculate Kd_{DOC} from our data points and the subsequent concentrations of

Hg and HgLA in order to apply (eq.1) to obtain the forward and back- reaction complexation rates as shown in Table 1 SI.

A second approach was used for MeHg. Considering that that only one dissolved MeHg data point is available before Hg accumulation in the cell, the DOC partition coefficient was thought to be underestimated. Consequently, a second assumption was used regarding the half-life for MeHg complexation to DOM available in the medium. To define Hg complexation rate constant with DOM the following equation was used:

$$k_1 = \frac{ln\,(2)}{\tau} \rightarrow k_8 = k_1 * K_D * DOC \qquad (2)$$

where k1 is the complexation rate constant (min^{-1}) and k8 is the desorption of Hg from DOM binding sites. This equation requires the definition of the half-life (τ). Since the need was to include a fast reaction. Hg binding with DOM half-life was defined as 5 min after the optimization of the best fit from a range of rates between 5 - 15 min.

Once the corresponding transfer and partitioning constants were defined. the corresponding equations describing change in Hg availability in the different studied phases were described (eq. 2) and computed using Scientific Python Development Environment Spyder 4.5.2 (Python 3.9.14). This software was used in order to numerically integrate the ordinary differential equations for the model of Hg distribution in the studied phases over time.

The summary of analytical solutions used for each forward and backward reaction rate can be found in Table 1 SI A. The definitive rates used for the model for adsorption were taken from the total particulate- dissolved Hg interaction, while internalization rates are obtained through the cell interior – phycosphere forward reaction rate. On the other hand, iHg efflux rate is the sum of the

rate of Hg released from the cell interior plus the cell interior–phycosphere back-reaction rate. Nevertheless, considering particulate MeHg concentrations had reached a plateau in the first 2 h and that only one data point is available within this period. the calculated adsorption rate constant was thought to be underestimated. Therefore, an optimization of rate iterations was used for the estimation of MeHg internalization and efflux rate. as well as for missing reaction rates for Hg-LA complexation.

Table 1 SI A. Summary of analytical solutions used for each forward and backward reaction rate

# k	Direction	iHg	MeHg
1	Diss → LA	$k_1 = \frac{[Hg]LA_1 - [Hg]LA_0}{[Hg]d_0 * (t_1 - t_0)}$	$k_1 = \frac{ln\,(2)}{\tau}$; $\tau = 5\ min$
2	Diss → Ads	$k_2 = \frac{[Hg]p_1 - [Hg]p_0}{[Hg]d_0 * (t_1 - t_0)} * 0.2$	$k_2 = \frac{[Hg]p_1 - [Hg]p_0}{[Hg]d_0 * (t_1 - t_0)} * 0.3$
3	LA → Ads	$k_3 = k_1 * \frac{1}{Kd_{DOC} * DOC}$	$k_3 = k_1 * \frac{1}{Kd_{DOC} * DOC}$
4	Ads → Int	$k_4 = \frac{[Hg]int_1 - [Hg]int_0}{[Hg]ads_0 * (t_1 - t_0)} +$ $k_2 \frac{[Hg]d_1}{[Hg]ads_1}$	Iterative optimization<br>Lowest MSE
5	Int → Ads	$k_5 = k_4 * \frac{1}{Kd_{int/ads} * POC}$	Iterative optimization<br>Lowest MSE
6	Ads → Diss	$k_6 = k_2 * \frac{1}{Kd_{POC} * POC}$	$k_6 = k_2 * \frac{1}{Kd_{POC} * POC}$
7	Ads → LA	$k_7 = k_3 * \frac{1}{Kd_{POC} * POC}$	$k_7 = k_3 * \frac{1}{Kd_{POC} * POC}$
8	DOM → Diss	$k_8 = k_1 * Kd_{DOC} * DOC$	$k_8 = k_1 * Kd_{DOC} * DOC -$ $k_3 + k_7$

Appendix B: Mercury diffusion

Metal-organism interactions have been represented in the past. e.g. by the biotic ligand model BLM[4]. Following our operational differences, we represent our Hg-Tiso interaction model as illustrated in the graphical abstract modified from Campbell (1995). In order to better define the amount of Hg available for internalization and speciation. the diffusional fluxes of iHg and MMHg from the bulk solution to the biological surface were computed. The diffusional flux of a substance through a boundary layer around a spherical cell in an unstirred bulk solution is described with the following equation:

$$J_{diff} = \frac{4\pi D(C_b - C_s)\left(\frac{r_d r_c}{r_d - r_c}\right)}{A} \tag{8}$$

where rc is the mean radius of the cell (cm); rd is the thickness of the unstirred boundary layer added to the radius of the cell (cm); D is the diffusion coefficient of the metal ($cm^2\ s^{-1}$); Cb and Cs are the metal concentrations in the bulk solution and at the algal surface ($mol\ cm^{-3}$). respectively; and A is the mean algal surface area (cm2) [5]. Diffusion coefficients were taken from Mills & Lobo (1989) and Gills et al. (1999).[5] while the rest of input data was taken from the results of each experiment; including supporting data.

The diffusional flux was found to be $2.6x10^{-15}\ mol\ cm^{-2}\ s^{-1}$ for iHg and $3.6x10^{-15}\ mol\ cm^{-2}\ s^{-1}$ for MMHg, while the iHg and MMHg was taken up at $1.1x10^{-18}\ mol\ cm^{-2}\ s^{-1}$ and $6.3x10^{-18}\ mol\ cm^{-2}\ s^{-1}$ respectively. The results indicate the flux of Hg that is able to diffuse through the outer layer, also known as phycosphere, where sorption and surface complexation take place at passive binding sites within the layer. in contrast with the flux of Hg internalized by the cell. The diffusional flux is higher by several orders of magnitude than the internalization Hg flux into the cell. Thus, the

internalization of Hg through the cell wall is not diffusion-limited. Hence. the diffusive flux of Hg to the cell surface or phycosphere can maintain the observed flux of Hg internalization. Moreover. even though MeHg diffusion to the phycosphere proved to be faster than iHg, the difference between the diffusion and uptake is higher for MeHg. Indicating that transference of MeHg from the phycosphere to the cell interior is also faster.

List of Figures

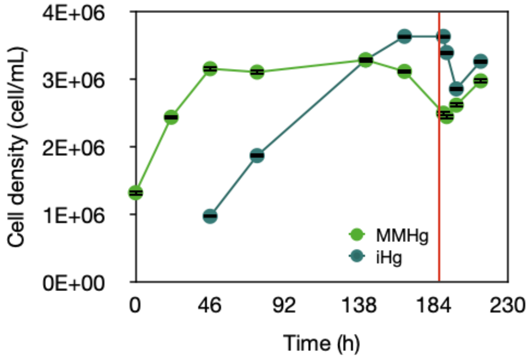

Figure 1 SI. Cell density over time. Red line corresponds to the start of the experiment.

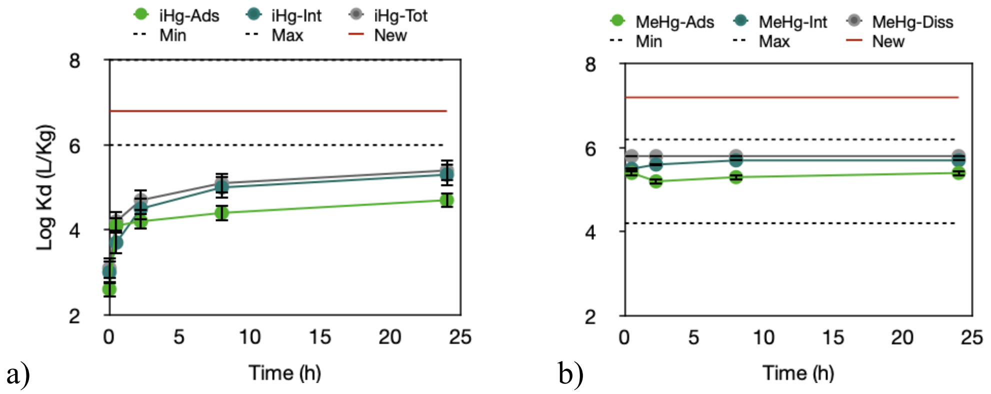

Figure 2 SI. Inorganic mercury a) and Methylmercury b) log partition kinetics associated with total particulate. adsorbed and absorbed phases with calculations based on SPM. Dotted lines for THg graph correspond to the range of Kd values in three ocean basins taken from Cui et al. (2021). while dotted lines in MeHg graph correspond to the range of Kd values described in Allison and Allison (2005) from experimental studies. Red lines represent the values for each Hg species. recomputed for SPM/open ocean Hg levels ratio.

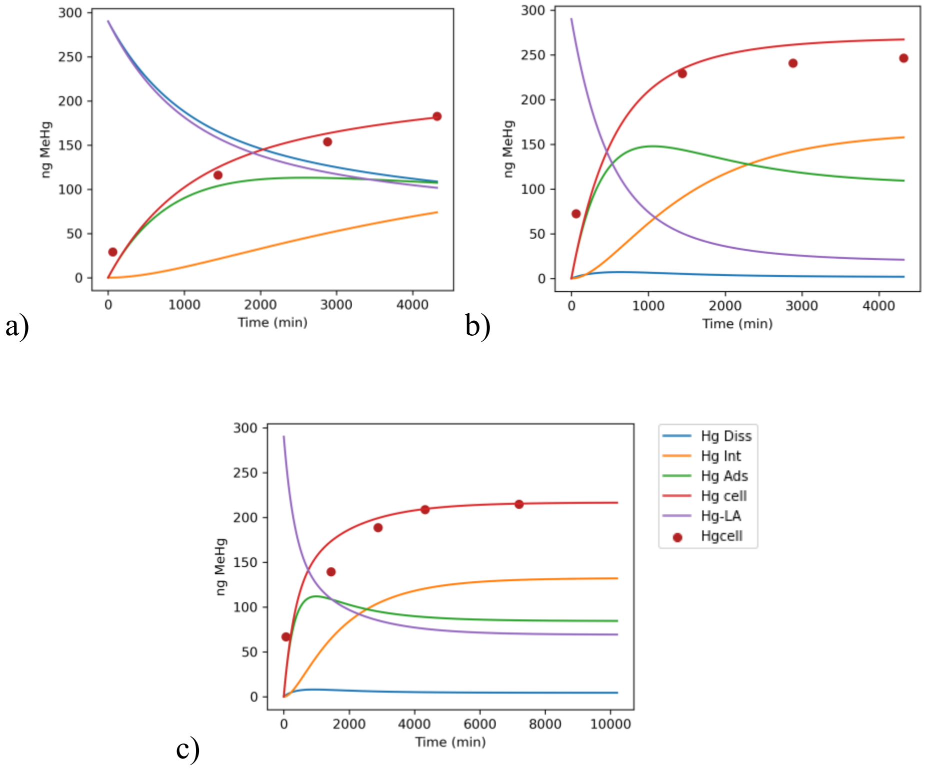

Figure 3 SI. MMHg cell accumulation inventories and numerical integration associated with total particulate (Hg-cell), adsorbed (Hg-ads) and internalized (Hg-int) phases for the 4-site three compartment model based on available mgC and MMHg exposure in each experiment for diatoms a), dinoflagellate b) and cyanobacterium c) from Lee and Fisher (2016).[6]

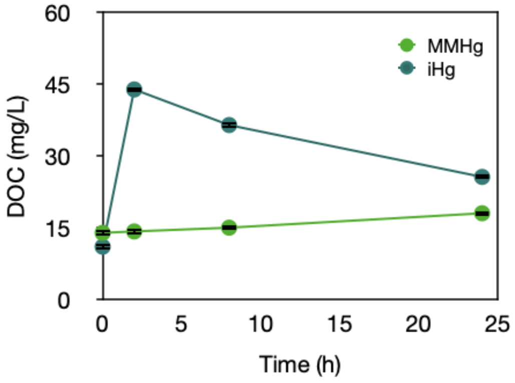

Figure 4 SI. Dissolved organic carbon concentrations measured during inorganic mercury a) and Methylmercury b) experiments.

List of Tables

Table 2 SI. Summary of mean square error (MSE) of modelled vs observed Hg in each compartment. showing the improvement of fit with added reservoirs.

		iHg	MeHg
Model MSE	Dissolved	0.27	0.10
	Adsorbed	0.070	0.055
	Internalized	0.52	0.017

Table 3 SI. Cellular Hg uptake rates constants (normalized to the recovered total Hg concentrations in the culture) expressed per cell (amole cell^{-1} h^{-1}), per calculated cell surface area (amole µm^{-2} h^{-1}). and per measured cell volume (amole µm^{-3} h^{-1}).

		*cell density**		*cell surface***		*cell volume****	
# k	*Direction*	***MeHg***	***iHg***	***MeHg***	***iHg***	***MeHg***	***iHg***
1	Diss → LA	2,73E+00	8,24E-01	5,32E-02	1,47E-02	7,89E-02	2,09E-02
2	Diss → Ads	6,48E-01	4,22E-02	1,26E-02	7,54E-04	1,88E-02	1,07E-03
3	LA → Ads	2,72E+00	1,47E+00	5,31E-02	2,63E-02	7,89E-02	3,74E-02
4	Ads → Int	1,14E+00	1,95E-01	2,23E-02	3,48E-03	3,30E-02	4,95E-03
5	Int → Ads	7,28E-01	4,20E-02	1,42E-02	7,50E-04	2,11E-02	1,07E-03
6	Ads → Diss	1,68E-02	1,30E-02	3,27E-04	2,31E-04	4,86E-04	3,29E-04
7	Ads → LA	1,87E-01	5,11E-01	3,65E-03	9,13E-03	5,42E-03	1,30E-02
8	LA → Diss	1,92E-01	4,61E-01	3,74E-03	8,23E-03	5,55E-03	1,17E-02
Total	Diss → Cell	4,10E+00	1,56E+00	8,00E-02	2,78E-02	1,19E-01	3,95E-02
Total	Cell → Diss	1,35E+00	1,51E+00	1,31E-13	6,40E-14	9,76E-02	3,84E-02

unit:*amole MeHg cell^{-1} h^{-1} nM^{-1} exposure; ** amole MeHg µm^{-2} h^{-1} nM^{-1} exposure; *** amole MeHg µm^{-3} h^{-1} nM^{-1} exposure

Table 4 SI. 4-site model transfer rate constants of Hg species accumulation for diatom, dinoflagellate and cyanobacterium on a biomass basis (mgC cell $^{-1}$ h^{-1}). The numbers of k's refer to the processes identified in Figure 1.

# k	*Direction*	*Diatom*	*Dinoflagellate*	*Cyanobacterium*
1	Diss → LA	5,55E-03	5,72E-04	1,44E-01
2	Diss → Ads	1,32E-03	1,36E-04	3,42E-02
3	LA → Ads	5,55E-03	5,71E-04	1,44E-01
4	Ads → Int	2,32E-03	2,39E-04	6,02E-02
5	Int → Ads	1,48E-03	1,53E-04	3,84E-02
6	Ads → Diss	3,42E-05	3,52E-06	8,85E-04
7	Ads → LA	3,81E-04	3,93E-05	9,88E-03
8	LA → Diss	3,90E-04	4,02E-05	1,01E-02

References

(1) Benoit, J. M.; Mason, R. P.; Gilmour, C. C.; Aiken, G. R. Constants for Mercury Binding by Dissolved Organic Matter Isolates from the Florida Everglades. *Science (80-.).* **2001**, *65* (24), 4445–4451.

(2) Bieser, J.; Amptmeijer, D.; Daewel, U.; Kuss, J.; Soerenson, A. L.; Schrum, C. The 3D Biogeochemical Marine Mercury Cycling Model MERCY v2.0; Linking Atmospheric Hg to Methyl Mercury in Fish. *Geosci. Model Dev. Discuss.* **2022**, No. October, 1–59.

(3) Onrubia, J. A. T.; Petrova, M. V; Puigcorbé, V.; Black, E. E.; Dufour, A.; Hamelin, B.; Buesseler, K. O.; Masqué, P.; Frederic, A. C.; Moigne, L.; Sonke, J. E.; Loeff, M. R. Van Der. Mercury Ex Port Flux in the Arctic Ocean Estimated from Th 234 : U 238 Disequilibrium.

(4) Campbell, P. G. C. Interactions between Trace Metals and Acuatic Organisms: A Critique of the Free-Ion Activity Model. John Wiley & Sons, Ltd 1995, pp 45–97.

(5) Le Faucheur, S.; Campbell, P. G. C.; Fortin, C.; Slaveykova, V. I. Interactions between Mercury and Phytoplankton: Speciation, Bioavailability, and Internal Handling. *Environ. Toxicol. Chem.* **2014**, *33* (6), 1211–1224. https://doi.org/10.1002/etc.2424.

(6) Lee, C. S.; Fisher, N. S. Methylmercury Uptake by Diverse Marine Phytoplankton. *Limnol. Oceanogr.* **2016**, *61* (5), 1626–1639. https://doi.org/10.1002/lno.10318.
